# Female genetic distribution bias in mitochondrial genome observed in Parkinson’s Disease patients in northern China

**DOI:** 10.1038/srep17170

**Published:** 2015-11-25

**Authors:** Qiaohong Chu, Xiaoguang Luo, Xiaoni Zhan, Yan Ren, Hao Pang

**Affiliations:** 1 School of Forensic Medicine, China Medical University, No.77 Puhe Road, Shenyang North New Area, Shenyang 110122, P.R. China; 2 Department of Neurology, 1st Affiliated Hospital of China Medical University, Shenyang 110001, P.R. China

## Abstract

Genetic polymorphisms associated with susceptibility to Parkinson’s disease (PD) have been described in mitochondrial DNA (mtDNA). To explore the potential contribution of mtDNA mutations to the risk of PD in a Chinese population, we examined the linkage relationship between several single nucleotide polymorphisms (SNPs) and haplotypes in mtDNA and PD. We genotyped 5 SNPs located on coding genes using PCR-RFLP analysis. A specific allele 10398G demonstrated an increased risk of PD (OR 1.30; 95% CI 0.95–1.76; *P* = 0.013). After stratification by gender, the increased risk appeared to be more significant in females (OR 1.91; 95% CI 1.16–3.16; *P* = 0.001). But the significance only appeared in females under Bonferroni correction. No significant differences were detected for other SNPs (T4336C, G5460A, G9055A, and G13708A). Individual haplotype composed of 4336T-5460G-9055G-10398A-13708G was found to be associated with protective effect regarding PD (*P* = 0.0025). The haplotypes 4336T-5460G-9055G-10398G-13708G and 4336T-5460G-9055G-10398A-13708G were more significantly associated in females (*P* = 0.0036 for risk and *P* = 0.0006 for protective effects). These data suggest that the A10398G and two haplotypes coupled with 10398A or 10398G are closely associated with susceptibility to PD in a northern Chinese population. This association demonstrated a female genetic distribution bias.

Mitochondria are cellular organelles that perform metabolic reactions necessary to generate energy as adenosine triphosphate (ATP). Mitochondria contain their own DNA in a single circular chromosome (mtDNA) and their own machinery for RNA and protein synthesis. The mitochondrial genome has 37 intronless genes that encode 13 subunits of the electron-transfer chain, 2 ribosomal RNAs, and 22 transfer RNAs^1^. Mutations in mtDNA could reduce the capacity to produce ATP, and this impairment in energy supply could affect neurons and other cell types. It has been well established that mtDNA mutations are responsible for several syndromes, such as Wolfram (DIDMOAD) syndrome, Leber hereditary optic neuropathy (LHON), and Leigh syndrome (LS)^2^,^3^,^4^. The mtDNA mutations involved in these severe syndromes dramatically affect mitochondrial function and frequently involve several genes (large deletions) or mitochondrial protein synthesis (e.g., mutations in mitochondrial rRNAs and tRNAs). These are commonly observed as familial diseases with non-Mendelian maternal transmission. In addition, common mtDNA polymorphisms could influence the risk of developing multifactorial neurodegenerative disorders.

Several reports have suggested that mitochondrial dysfunction could be involved in neurodegenerative diseases, such as Parkinson’s disease (PD) and Alzheimer disease (AD)^5^,^6^,^7^. A moderate impairment of complex I (the first system in the electron transport chain) has been demonstrated in patients with PD, and the substantia nigra and platelets of PD patients have reduced complex I activity^8^,^9^,^10^,^11^. In animal models, inhibition of complex I by several substances leads to selective degeneration of dopaminergic neurons, a hallmark of PD^12^. With disruption of the gene for mitochondrial transcription factor A (*Tfam*) in DA neurons, a conditional knockout mice (termed MitoPark mice) was created. The knockout mice have reduced mtDNA expression and developed respiratory chain deficiency in midbrain DA neurons, leading to a parkinsonism phenotype^13^,^14^. mtDNA encodes seven of the protein subunits of complex I, and genetic variation within the mitochondrial genome could contribute to the risk of developing PD^15^. In Asia, particularly China, genetic research on mtDNA polymorphisms related to PD is lacking^16^. Several mitochondrial single nucleotide polymorphisms (SNPs) in PD patients and controls have been genotyped^17^,^18^,^19^,^20^. According to the data, several of the mtSNPs evaluated could significantly contribute to the risk of developing PD.

T4336C in *tRNAGln*, G5460A in *ND2*, A10398G in *ND3*, and G13708A in *ND5* are complex I-related gene polymorphisms, while G9055A in *ATP6* is a polymorphic locus related to a gene that encodes ATP synthetase. Results from association studies evaluating complex I and electron transport chain gene polymorphisms and contribution to PD risk are disparate. In this study, we genotyped 322 PD patients and 332 healthy controls in northern China for 5 mitochondrial SNPs using polymerase chain reaction (PCR) followed by restriction fragment length polymorphism (RFLP) analysis. Allele and haplogroup frequencies were compared between patients and controls. Associations between A10398G and PD and gender stratification were also evaluated.

## Materials and Methods

### Subjects and sample collection

A total of 322 ethnic Han Chinese PD patients from northern China were included in the study (mean age ± SD 59.65 ± 12.86; range 40 to 86 years; 173 men, 149 women). Patients were diagnosed with idiopathic PD by movement disorder neurologists at the First Affiliated Hospital of China Medical University in the Liaoning province in China. All patients met the criteria for a clinical diagnosis of PD, presenting with at least two of the three cardinal signs of PD (e.g., tremor, rigidity, and bradykinesia) and had a positive response to levodopa therapy. A total of 332 unrelated control participants matched for ethnicity, age, and gender were recruited from the local community (mean age ± SD 58.97 ± 13.53; range 40 to 95 years; 221 men, 111 women). Control participants were healthy and had not been diagnosed with neurodegenerative diseases. Fewer female controls were recruited due to limited availability. The study protocol was approved by the Ethics Committee on Human Research, the China Medical University. The study procedures were performed in accordance with the tenets of the Declaration of Helsinki. Informed consent was obtained from all study participants. Peripheral blood samples were collected from participants, and DNA was extracted from leukocytes using the sodium dodecyl sulfate-proteinase K phenol-chloroform method.

### Mismatched primer design

**Figure 1:**
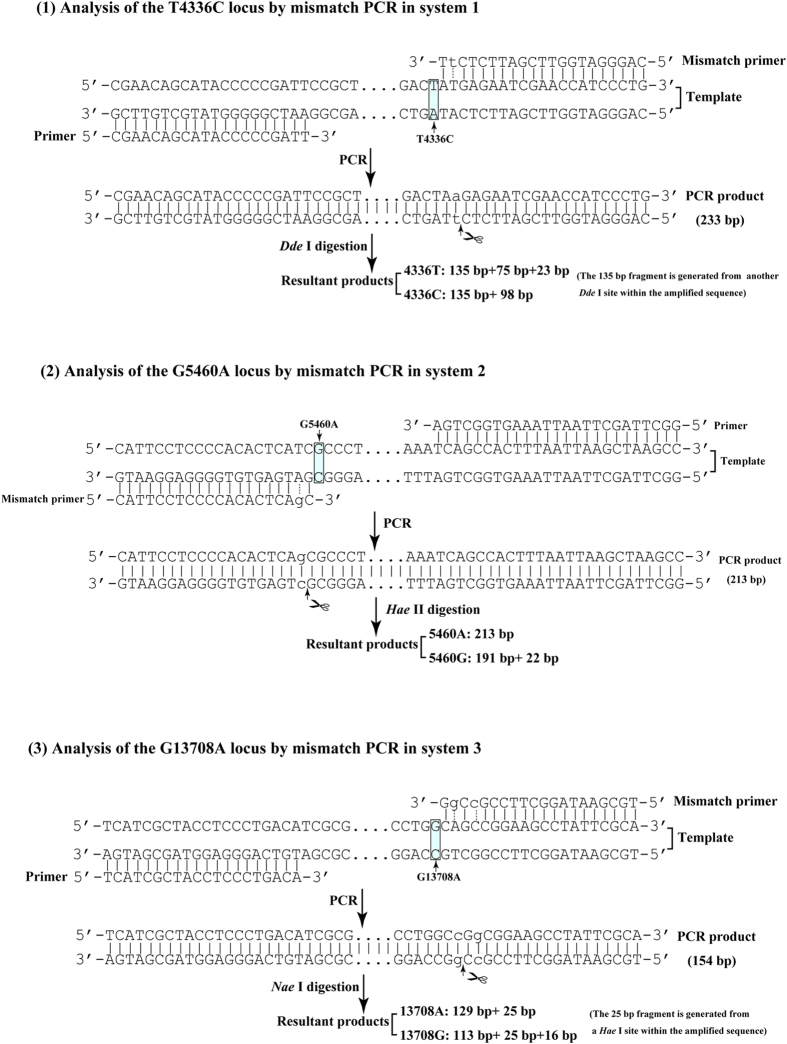
Analyses of the three SNPs by mismatch PCR assay. DNA sequences around the polymorphic sites and of the primers binding in the three SNPs were aligned. The mismatch base pairs were shown in dotted lines. The arrows plus scissors stand for the cutting sites for the restriction enzymes. The fragment sizes of PCR and resultant products digested by different restriction enzymes were included in the diagram.

To simultaneously genotype the G5460A, G9055A, and G13708A loci, we synthetically generated *Hae* II and *Nae* I restriction endonuclease sites in the amplified products of mitochondrial G5460A and G13708A loci using mismatched PCR primers based on published “revised Cambridge Reference Sequence (rCRS)” (http://www.mitomap.org) (e.g., a native *Hae* II restriction endonuclease site near the 9055G allele). Similarly, we synthetically generated a *Dde* I restriction endonuclease site in the amplified product of the mitochondrial T4336C locus using mismatched PCR primers to attain multiplexed genotyping with the A10398G locus, as there is a native *Dde* I restriction endonuclease site near the 10398G allele. The primers used for SNP detection are shown in Table 1 and the analyses involving in the mismatch PCR are shown in Fig. 1.

### mtDNA SNP genotyping

A total of 5 mitochondrial DNA fragments were amplified using PCR. Genotyping was performed via three separate PCR amplifications. In system 1, both the T4336C and A10398G polymorphisms were amplified using the primers and conditions described as follows. In system 2, the polymorphisms amplified were G5460A and G9055A. In system 3, only G13708A polymorphism was amplified. Each reaction in both systems 1 and 2 contained approximately 50–100 ng of genomic DNA, 2×powerTaq PCR MasterMix (Bioteke, Beijing, China), and the indicated primers at the concentrations listed in Table 1 in a final volume of 20 μl. PCR for system 3 was conducted in 20 μl *rTaq* buffer containing approximately 50–100 ng of genomic DNA, 1 U *rTaq* DNA polymerase (TaKaRa, Dalian, China), and 200 mM dNTP. Two multiplex and one single PCR were performed under the following cycle conditions: initial denaturation of 94 °C for 1 min, followed by 30 cycles (for system 1) or 35 cycles (for system 2 and 3) of 94 °C, denaturation for 30 s, 55 °C annealing for 30 s, and 72 °C elongation for 30 s, followed by a final extension at 72 °C for 1 min.

**Figure 2:**
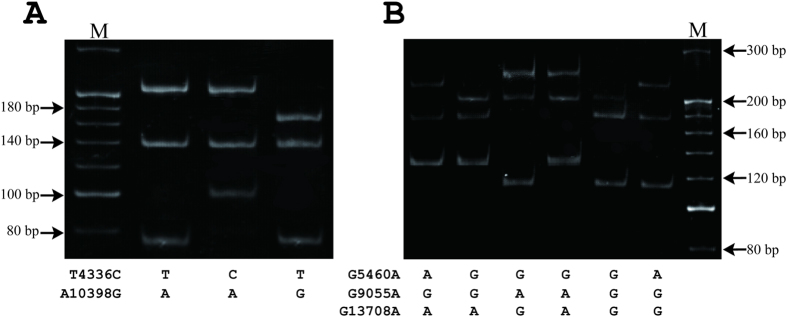
Electrophoretic pattern of mismatched multiplex polymerase chain reaction (PCR)–restriction fragment length polymorphism (RFLP) in five single nucleotide polymorphisms (SNPs). M lanes show a 20 bp DNA molecular size ladder ranging from 80 to 300 bp. (**A**) Electrophoretic patterns for SNPs T4336C and A10398G were exhibited and alleles were indicated under each lane. (**B**) Electrophoretic patterns for SNPs G5460A, G9055A and G13708A were exhibited and alleles were indicated under each lane. bp = base pairs.

The genotype corresponding to each polymorphism was determined through RFLP analysis (Table 1). For restriction enzyme digestion, 1 μl of each PCR product was digested with the appropriate restriction enzyme. Amplification products from system 1 and 0.25 μl *Dde* I (TaKaRa, Dalian, China) were mixed in 10 μl TaKaRa K buffer. Amplification products from systems 2 and 3 and 0.25 μl *Hae* II and *Nae* I (TaKaRa, Dalian, China) were mixed in 10 μl TaKaRa M buffer. PCR products were incubated at 37 °C for one hour. Each digestion yielded several common fragments. Digestions were separated on 6% polyacrylamide gel, and fragments were visualized after ethidium bromide staining (Fig. 2). Nucleotides in the mtDNA were numbered according to the rCRS.

### Statistical analysis

Statistical analyses were performed using Haploview version 2.0 software. Allele and haplotype frequencies between patients and controls were compared by the χ^2^ test. Odds ratios (ORs) and 95% confidence intervals (CIs) were also calculated to assess the odds of carrying each allele in patients compared with controls. Haplotypes were generated by Haploview and PowerMarker (version 3.25). Pair-wise linkage disequilibrium (LD) was computed using Haploview, while multi-locus linkage disequilibrium was computed using PowerMarker. For all association tests, a raw *P* value of less than 0.05 was considered as nominally significant, which was subjected to Bonferroni correction to account for multiple comparison problems. In this study, the significance threshold for single SNP tests was set as 0.01 (0.05/5) since 5 SNPs were included in the association analyses. Similarly, the significance threshold for haplotype analysis was set as 0.0045 (0.05/11) since 11 haplotypes were included in the association analyses^21^.

## Results

### Genotyping five mitochondrial SNPs

The use of a mismatched PCR primer to synthetically create a restriction site in the amplified product makes it possible to solve many common polymorphisms that fail to create or remove any restriction sites (as shown in Fig. 1). The prerequisites for potentially applicable multiplex mismatch PCR assay are an identical restriction site introduced and close melting temperature generated from the designed primers. Following DNA sequencing confirmation, we performed mismatched multiplex PCR amplification and RFLP analysis. The results showed that the mismatched sequences containing *Hae* II, *Dde* I or *Nae* I recognition sites were specifically amplified at all loci. The sizes of the amplified PCR products were 233 base pairs (bp) (T4336C), 205 bp (A10398G), 213 bp (G5460A), 232 bp (G9055A), and 154 bp (G13708A), respectively (as shown in Table 1 and Fig. 1). PCR products were separated by electrophoresis after digestion with *Dde* I in system 1 or *Hae* II and *Nae* I in systems 2 and 3. Digested fragments for allele determination are listed in Table 1 and Fig. 1. The genotypes of T4336C and A10398G loci in system 1 were shown in Fig. 2A. The ones of G5460A, G9055A and G13708A in systems 2 and 3 were in Fig. 2B.

Fragments shorter than 40 bp were migrated out of the gel under electrophoretic conditions. This novel mismatched multiplex PCR-RFLP assay demonstrated successful genotype identification of the five SNPs.

### A10398G in the *ND3* gene showed significant association with PD

Allelic frequency distribution of the five SNPs is shown in Table 2. Subsequent association analyses of the five SNPs showed that genotype distribution of A10398G in the *ND3* gene was significantly different between the two groups (Table 3). Based on current data, our results suggest that the 10398G allele is a risk factor for PD in individuals from northern China (*P* = 0.013) (Table 3), but it couldn’t survive the Bonferroni correction. However, no associations were observed for other polymorphisms (Tables 2 and 3).

### Female genetic distribution bias

We performed gender stratification to understand associations between mitochondrial SNPs and PD. As expected, associations between the T4336C, G5460A, G9055A, and G13708A loci and PD were not observed in male or female populations (Table 3). However, we found a significant difference in the A10398G locus in the female cohort (*P* = 0.001), demonstrating that 10398G is a stronger risk factor for PD in women. In contrast, no associations were found in the male cohort (*P* = 0.984) (Table 3).

**Figure 3:**
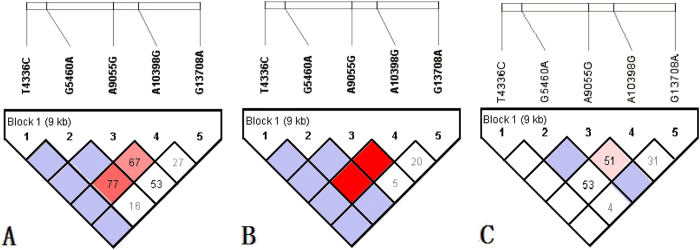
G5460A and A10398G and G9055A and A10398G were in linkage disequilibrium (LD). (**A**–**C**): Results from linkage analysis conducted for the SNPs of total population, female population and male population, respectively.

We entered the data into Haploview 2.0 and PowerMarker 3.25 software and obtained eleven haplotypes with five SNP loci (Table 4). Results from linkage analysis conducted for the SNPs showed that G5460A and A10398G, and G9055A and A10398G were both in linkage disequilibrium (both LD >0.6, Fig. 3). Meanwhile, the multi-loci linkage analysis showed that the five loci were in linkage disequilibrium (*P* < 0.05). Therefore, we further analyzed possible haplotype associations with PD in the population investigated. As shown in Table 4, haplotype frequency for 4336T-5460G-9055G-10398A-13708G revealed significant differences between case and control groups (*P* = 0.0025), suggesting that the haplotype may be protective due to 10398A. After gender stratification, significant associations in both 4336T-5460G-9055G-10398G-13708G and 4336T-5460G-9055G-10398A-13708G haplotypes were observed in the female cohort (*P* = 0.0036 and *P* = 0.0006, respectively). However, in the male cohort, there was no difference observed among the haplotypes between case and control groups. These results suggest that there is a stronger linkage relationship between PD patients and controls based on mtDNA polymorphism analysis, with a female genetic distribution bias in the northern Chinese population.

## Discussion

Sporadic PD has a complex etiology, and it is widely considered that genetic factors act independently or in concert with other genetic and/or environmental factors. Because mitochondrial dysfunction has been involved in the expression of PD, mtDNA polymorphisms could influence the risk of developing PD, either directly or through interaction with other genes or environmental toxins. To test this hypothesis, we genotyped five mtDNA SNPs in a Chinese population. The 9055A allele on *ATP6* demonstrated a protective effect in women of European ancestry in a previous study^22^, but we failed to replicate this finding in a Chinese population. The T4336C polymorphism in the *tRNA*^*gln*^ gene in mtDNA has been linked to both AD and PD^16^,^23^,^45^. Both positive and negative results regarding that locus have been subsequently reported. A meta-analysis of individual gene polymorphisms supported a significant association between the T4336C polymorphism and PD^7^. Similarly, a higher risk for PD among women with the mitochondrial 4336C allele in a Spanish population was described^8^,^19^, but this effect was not observed in male patients. In contrast, Mayr-Wohlfart *et al.* was not able to detect a difference in the frequency of the T4336C mutation between patients and controls in Germany^24^. The rarity of *tRNA*^*Gln*^ 4336C in a central Virginia population precluded researchers from postulating that this polymorphism protects against PD^11^. In the present study, we found one female PD patient with the 4336C allele. Our data adequately support the hypothesis that the *tRNA*^*Gln*^ 4336C variant does not meaningfully contribute to PD in individuals in northern China. The G5460A polymorphism located on the *ND2* gene was detected in the form of heteroplasmy in several brains of patients with idiopathic PD^25^. In German controls, the 5460A frequency (5/77; 6.5%) was more than double the central Virginia 5460A control rate of 2.8%. While the G5460A polymorphism was very rare in the central Virginia PD cohort^11^, no mutation in the gene was detected in a population from Spain^8^. In the present study, we did not find heteroplasmic G5460A in whole blood samples from idiopathic PD patients, although allele frequencies on the G5460A locus were not low in both patients and controls in the Chinese population studied. Similarly, no association between G5460A and PD was detected. The G13708A polymorphism in the *ND5* gene has been involved in several mitochondrial haplogroups^8^,^22^. Previously, a report on PD patients of European ancestry showed that the 13708A allele was protective in a group of patients ≥70 years of age^22^. However, we did not find an association between the G13708A locus and PD in the northern Chinese population. The results and comparisons from these 4 loci on mtDNA suggest that differences in frequency distribution are present in different populations and that the 4 loci do not represent any risk or susceptibility factors for PD in the northern Chinese population.

Interestingly, the polymorphic A10398G locus in the *ND3* gene has been implicated in the etiology of several diseases. The substitution of threonine (10398A) for alanine (10398G) is relatively non-conservative from the perspective of chemical properties. Threonine can form hydrogen bonds cross-linking protein chains and moreover is susceptible to numerous posttranslational modifications. The methyl group of alanine is non-reactive and is thus almost never directly involved in protein function^26^. Although no structural or apoptosis/proliferation data are currently available in 10398A and 10398G cells, one current hypothesis for A10398G proposes that increase in the production of reactive oxygen species (ROS) may arise as a consequence of altered complex I function. This is especially important as increased free radical generation and local oxidative injury facilitate neoplastic transformation and metastasis^44^. 10398A was reported to increase the risk of AD in men^27^, invasive breast cancer in African-American women^44^, and prostate cancer in African-American men^46^. Also, 10398A is reportedly associated with an increased risk of bipolar disorder^28^, and 10398G has been associated with longevity^29^. In addition, the A10398G SNP was found in 90% of LHON patients tested and seems to be a genetic factor in type 2 diebetes mellitus (T2DM)^30^,^31^. In particular, association between A10398G and PD showed conflicting results. van der Walt and colleagues reported a reduced frequency of this polymorphism in Caucasian PD patients, suggesting a protective effect^22^, and an inverse association was confirmed in a subsequent study in Spanish patients^19^. A previous study found no association of the 10398G variant with the risk of PD in a subset of Caucasian subjects^15^, and a later study in Italian subjects similarly detected no association of this variant with PD^32^. It has been postulated that the A10398G polymorphism may play a role in the pathophysiology of these complex diseases by affecting mitochondrial matrix pH and intracellular calcium dynamics^33^. The results suggest that the A10398G locus in mtDNA is complex in its influence of different diseases, even within single diseases. Recent reports in a Chinese population suggest that the *ND3* gene and A10398G may not be an independent risk factor for breast cancer^34^,^35^, whereas another study showed that A10398G seems to be a genetic factor in T2DM in a Chinese Han population. The present study, which included PD patients and controls from a Chinese population, also identified a significant association between the 10398G variant and the risk of PD, similar to results reported in the Basque population in Spain, but in contrast to the European ancestry results described by van der Walt *et al.* Variability in reported associations between the A10398G locus and PD could be related to distinct subject populations across studies. It is implied that the locus plays an amphoteric role in interactions between mtDNA polymorphisms, mtDNA SNPs, and nuclear DNA, or between the polymorphism and environmental factors. Thus, further studies are needed to determine if this polymorphism influences the risk of PD in certain subsets of PD patients and acts as a coordinator involved in oxidative phosphorylation in the mitochondria.

Evidence to support the possibility that mtDNA variants may play a role in PD is the reported bias towards maternal inheritance of PD^36^,^37^, though a maternal bias has not been detected in all studies^38^,^39^. A multigenerational family was reported to have maternally inherited PD associated with a mitochondrial complex I defect in transmitochondrial hybrid cells (cybrids) created from affected family members^40^. van der Walt *et al.* first described that the protective effect of allele 10398G should be stronger in women than men with PD of European ancestry^22^. However, the possibility of a maternal inheritance bias in 168 multiplex PD families from New Jersey in the United States was not detected by analyzing the frequency of the 10398G complex I gene polymorphism in PD patients compared to controls^41^. Interestingly, we observed a significantly increased frequency of 10398G in women with PD compared to controls, but this effect was not present in male patients. The argument contained herein represents the opposite point of view from a previous report^22^. Differences in methodologies and patient populations may contribute to these disparate results. Mitochondrial genes are passed from mothers to their offspring, but not from fathers to offspring. Therefore, to the degree that heritable mitochondrial genetic factors influence the risk of PD, there should be a greater risk of PD among the offspring of women with PD compared to offspring of men with PD. Thus, further investigation of PD penetrance from both mothers and fathers with PD and carriers of the 10398G allele to their offspring might yield more information as to whether there is maternal inheritance bias or not.

Mitochondrial haplogroup clusters have been reported to be associated with both increased and decreased risk of PD^17^,^20^,^42^,^43^. A10398G characterizes the European haplogroup I, J, and K and Asian-specific super haplogroup M^44^. Because there is no evidence of major ethnic biases in the prevalence of PD, the very high frequency of 10398G in non-European populations makes it unlikely that this polymorphism confers a significant association with PD and instead, it is likely that it acts as an important contributor in cooperation with other mtDNA SNPs. Based on analysis of T4336C, G5460A, G9055A, A10398G and G13708A polymorphisms, we observed eleven possible haplotypes in this study. 4336T-5460G-9055G-13708G and 10398A or G could be jointly involved in influencing the risk of PD due to linkage disequilibrium between G5460A and A10398G as well as G9055A and A10398G, suggesting that compound genotypes might prove potentially relevant. Similarly, after gender stratification, the positive association with PD was only found in female cohorts in the population investigated. Thus, female genetic distribution bias on mtDNA was further supported based on the haplotypes. Additional research involving more SNPs in the ND genes of complex I or complete sequencing of the mtDNA in these cases are needed to confirm or refute these findings.

In contrast with several previous studies from other populations, we observed an increased risk for PD among carriers of 10398G. Moreover, women with 10398G allele had an increased risk for PD in the northern Chinese population. In addition, the haplotype 4336T-5460G-9055G-10398A-13708G had a protective effect for PD. Interestingly, two haplotypes 4336T-5460G-9055G-13708G and 10398A or 10398G had conflicting effects for the risk of developing this common neurodegenerative disorder, implying that female genetic distribution bias on mtDNA may occur in PD patients in northern Chinese population.

## Conclusions

In short, our data strongly suggest that the A10398G SNP and two haplotypes coupled with the 10398A or 10398G alleles are closely associated with susceptibility to PD in a northern Chinese population driven by a female genetic distribution bias.

## Additional Information

**How to cite this article**: Chu, Q. *et al.* Female genetic distribution bias in mitochondrial genome observed in Parkinson's Disease patients in northern China. *Sci. Rep.* **5**, 17170; doi: 10.1038/srep17170 (2015).

## Figures and Tables

**Table 1:** mtDNA polymorphism analyzed through digestion with a restriction enzyme.

Polymorphic locus	Primers (5’→3’)	Concentration (μM)	Restriction enzyme	Allele size (base pairs)
T4336C	CGAACAGCATACCCCCGATT	0.25	*Dde* I	T:135 + 75 + 23
	CAGGGATGGGTTCGATTCTCtT^a^			C:135 + 98
G5460A	CATTCCTCCCCACACTCAgC^a^	0.25	*Hae* II	A:213
	GGCTTAGCTTAATTAAAGTGGCTGA			G:191 + 22
G9055A	CGTACGCCTAACCGCTAACA	0.025	*Hae* II	A:232
	AGGCATGTGATTGGTGGGTC			G:172 + 60
A10398G	CCTAAGTCTGGCCTATGAGTGAC	0.25	*Dde* I	A:205
	TAGGGAGGATATGAGGTGTGAGCG			G:165 + 40
G13708A	TCATCGCTACCTCCCTGACA	0.25	*Nae* I	A:129 + 25
	TGCGAATAGGCTTCCGcCgG^a^			G:113 + 25+ 16

^a^Small letters represent the mismatched bases to artificially introduce endonuclease sites.

**Table 2:** Allelic distribution of each polymorphism in control and affected subjects.

SNP locus	Affected number (%)			Control number (%)			
	Total	Female	Male	Total	Female	Male	Allele frequency
4336C	1 (0.3)	1 (0.6)	0 (0.0)	0 (0.0)	0 (0.0)	0 (0.0)	0.002
4336T	321 (99.7)	148 (99.4)	173 (100.0)	332 (100.0)	111 (100.0)	221 (100.0)	0.998
5460A	11 (3.4)	6 (4.0)	5 (2.9)	8 (2.4)	4 (3.6)	4 (1.8)	0.029
5460G	311 (96.6)	143 (96.0)	168 (97.1)	324 (97.6)	107 (96.4)	217 (98.2)	0.971
9055G	306 (95.0)	143 (96.0)	163 (94.2)	314 (94.6)	107 (96.4)	207 (80.1)	0.948
9055A	16 (5.0)	6 (4.0)	10 (5.8)	18 (5.4)	4 (3.6)	14 (19.9)	0.052
10398G	186 (57.8)	96 (64.4)	90 (52.0)	168 (50.6)	54 (48.6)	114 (51.6)	0.541
10398A	136 (42.2)	53 (35.6)	83 (48.0)	164 (49.4)	57 (51.4)	107 (48.4)	0.459
13708A	24 (7.5)	8 (5.4)	16 (9.2)	17 (5.1)	5 (4.5)	12 (5.4)	0.063
13708G	298 (92.5)	141 (94.6)	157 (90.8)	315 (94.9)	106 (95.5)	209 (94.6)	0.937

**Table 3:** Association analysis of five mtDNA SNPs in the northern Chinese cohort.

	SNP	T4336C	G5460A	G9055A	A10398G	G13708A
Total	Associated allele	C	A	G	G	A
	χ^2^	2.078	1.216	0.118	6.235	3.129
	*P*	0.149	0.270	0.732	0.013	0.077
	OR (95% CI)	∞ (0,∞)	1.43 (0.57, 3.60)	1.10 (0.55, 2.19)	1.30 (0.95, 1.76)	1.49 (0.79, 2.82)
Female	Associated allele	C	A	A	G	A
	χ^2^	1.639	0.206	0.206	10.592	0.200
	*P*	0.201	0.650	0.650	0.001	0.655
	OR (95% CI)	∞ (0,∞)	1.12 (0.31, 4.06)	1.12 (0.31, 4.06)	1.91 (1.16, 3.16)	1.20 (0.38, 3.74)
Male	Associated allele	−	A	G	G	A
	χ^2^	NaN^a^	1.415	0.001	0	4.459
	*P*	0.0	0.234	0.975	0.984	0.0547
	OR (95% CI)	−	1.39 (0.37, 5.26)	1.29 (0.55, 2.97)	1.02 (0.68, 1.74)	1.71 (0.79, 3.74)

^a^Not a number.

**Table 4:** The eleven haplotypes defined by five mtDNA SNPs and their associations in case-control groups.

Haplotype					Frequency			χ^2^	*P* value	Frequency			χ^2^	*P* value	Frequency			χ^2^	*P* value
4336	5460	9055	10398	13708	Total	Case	Control			Female	Case	Control			Male	Case	Control		
T	G	G	G	G	0.483	0.508	0.459	3.057	0.0804	0.515	0.570	0.441	8.48	0.0036	0.462	0.453	0.468	0.175	0.6757
T	G	G	A	G	0.375	0.333	0.414	9.173	0.0025	0.358	0.295	0.441	11.826	0.0006	0.386	0.366	0.401	0.981	0.3221
T	A	G	G	G	0.024	0.031	0.018	2.363	0.1242	0.038	0.040	0.036	0.062	0.8039	0.015	0.023	0.009	2.623	0.1053
T	G	A	A	G	0.041	0.040	0.042	0.02	0.8884	0.035	0.034	0.036	0.023	0.8784	0.046	0.047	0.045	0.01	0.9221
T	G	G	G	A	0.023	0.031	0.015	3.779	0.0513	0.023	0.034	0.009	3.401	0.0652	0.023	0.029	0.018	1.061	0.3031
T	G	G	A	A	0.037	0.040	0.033	0.515	0.4728	0.023	0.013	0.036	2.886	0.0894	0.046	0.064	0.032	4.673	0.0506
T	G	A	G	G	0.009	0.006	0.012	1.202	0.2729	—	—	—	—	—	0.015	0.012	0.018	0.528	0.4675
T	A	G	A	G	0.003	0.003	0.003	0.001	0.9707	—	—	—	—	—	0.005	0.006	0.005	0.066	0.7975
C	G	G	A	G	0.002	0.003	0.000	2.078	0.1494	0.004	0.007	0.000	1.496	0.2213	—	—		—	—
T	A	G	G	A	0.002	0.000	0.003	1.931	0.1647	—	—	—	—	—	0.003	0.000	0.005	1.553	0.2126
T	G	A	A	A	0.002	0.003	0.000	2.078	0.1494	0.004	0.007	0.000	1.496	0.2213	—	—		—	—
